# Antitumor Activity of Abnormal Cannabidiol and Its Analog O-1602 in Taxol-Resistant Preclinical Models of Breast Cancer

**DOI:** 10.3389/fphar.2019.01124

**Published:** 2019-09-27

**Authors:** Andrea Tomko, Lauren O’Leary, Hilary Trask, John C. Achenbach, Steven R. Hall, Kerry B. Goralski, Lee D. Ellis, Denis J. Dupré

**Affiliations:** ^1^Department of Pharmacology, Faculty of Medicine, Dalhousie University, Halifax, Canada; ^2^Aquatic and Crop Resource Development Research Center, National Research Council of Canada, Halifax, Canada; ^3^College of Pharmacy, Faculty of Health, Dalhousie University, Halifax, Canada

**Keywords:** cannabinoid, taxol, paclitaxel, breast cancer, apoptosis, cell migration, G protein–coupled receptor, zebrafish

## Abstract

Cannabinoids exhibit anti-inflammatory and antitumorigenic properties. Contrary to most cannabinoids present in the *Cannabis* plant, some, such as O-1602 and abnormal cannabidiol, have no or only little affinity to the CB_1_ or CB_2_ cannabinoid receptors and instead exert their effects through other receptors. Here, we investigated whether the synthetic regioisomers of cannabidiol, abnormal cannabidiol, and a closely related compound, O-1602, display antitumorigenic effects in cellular models of breast cancer and whether it could reduce tumorigenesis *in vivo*. Several studies have shown the effects of cannabinoids on chemotherapy-sensitive breast cancer cell lines, but less is known about the antitumorigenic effects of cannabinoids in chemotherapy-resistant cell lines. Paclitaxel-resistant MDA-MB-231 and MCF-7 breast cancer cell lines were used to study the effect of O-1602 and abnormal cannabidiol on viability, apoptosis, and migration. The effects of O-1602 and abnormal cannabidiol on cell viability were completely blocked by the combination of GPR55 and GPR18-specific siRNAs. Both O-1602 and abnormal cannabidiol decreased viability in paclitaxel-resistant breast cancer cells in a concentration-dependent manner through induction of apoptosis. The effect of these cannabinoids on tumor growth *in vivo* was studied in a zebrafish xenograft model. In this model, treatment with O-1602 and abnormal cannabidiol (2 µM) significantly reduced tumor growth. Our results suggest that atypical cannabinoids, like O-1602 and abnormal cannabidiol, exert antitumorigenic effects on paclitaxel-resistant breast cancer cells. Due to their lack of central sedation and psychoactive effects, these atypical cannabinoids could represent new leads for the development of additional anticancer treatments when resistance to conventional chemotherapy occurs during the treatment of breast and possibly other cancers.

## Introduction

*Cannabis* has been used as a medicine throughout history to treat a variety of diseases. Recently, the medicinal use of cannabis and cannabinoids has gained general acceptance. However, the full therapeutic potential and efficacy of cannabis and compounds derived from it is still being elucidated for specific disease states. Phytocannabinoids are produced by the plant *Cannabis* and target both the endocannabinoid system and other biological pathways. This allows them to exert a wide array of effects both on the central nervous system and peripheral immune, cardiovascular, digestive, reproductive, and ocular systems (Chanda et al., 2019, Pertwee et al., 2010). Commercially available cannabinoids, such as dronabinol, nabilone, and others, are approved for the treatment of cancer-related side effects such as nausea and vomiting (Steele et al., 2019). Cannabinoids have also been shown to exhibit antitumorigenic properties in various preclinical cancer models (McAllister et al., 2015; Ladin et al., 2016; Blasco-Benito et al., 2018).

It is estimated that approximately 12% of women will develop breast cancer at one time during their lives (Harbeck and Gnant, 2017). While breast cancer mortality rates have declined because of improved therapies and early diagnosis, metastatic breast cancer (the primary cause of breast cancer mortality) is expected to develop in 20% to 30% of women with early breast cancer and remains incurable with a median 5-year survival of 25% (Morris et al., 2009; Rivera and Gomez, 2010; Cardoso et al., 2017). Although estrogen receptor (ER)– and progesterone receptor (PR)–positive (ER^+^/PR^+^) breast cancers are associated with a higher response rate to current therapies, innate and acquired resistance can occur, which represent a significant treatment challenge due to the likelihood of cancer recurrence and dissemination to other organs. Similarly, resistance can also be observed in other types of breast cancers, like the HER2^+^ subset, and triple-negative breast cancers. Triple-negative breast cancer shows the worst prognosis with aggressive proliferation, migration, and invasion abilities. Although some patients with this subtype respond well to chemotherapy, many others do not respond. While metastatic breast cancer often responds well to initial treatments, the eventual development of multidrug resistance is expected and presents a major challenge for effective long-term treatment (Longley and Johnston, 2005; Coley, 2008; Marquette and Nabell, 2012; Robey et al., 2018). Consequently, development of new agents with low susceptibility to common drug resistance mechanisms (e.g., enhanced drug efflux *via* ATP-binding cassette transporters) is needed to ameliorate the anticancer response and possibly increase survival rates (Nobili et al., 2012).

**Figure 1:**
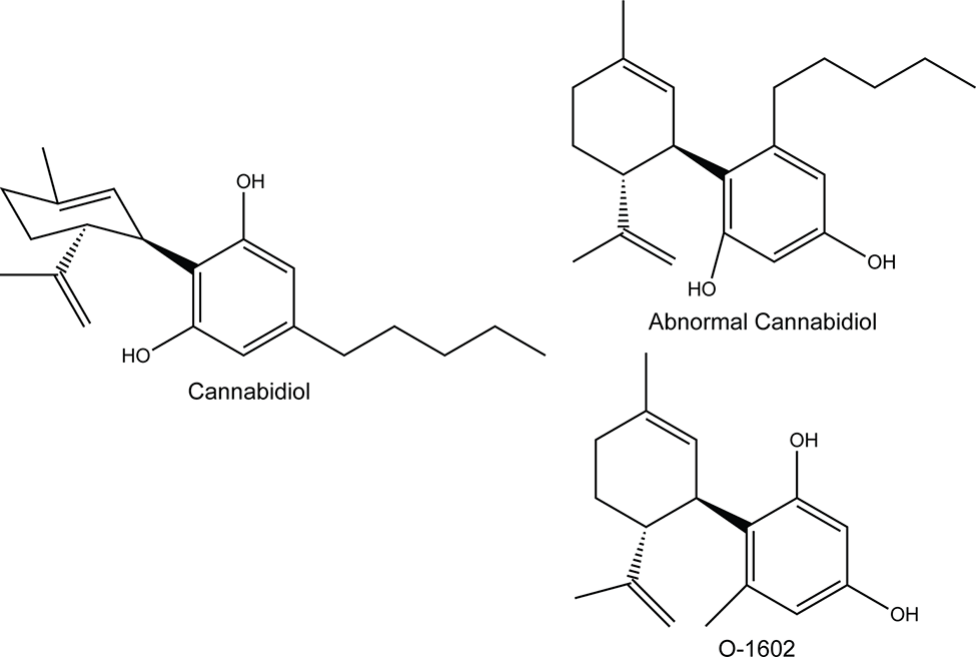
Structure of the compounds used in this study; O-1602 and abnormal cannabidiol, compared to cannabidiol.

It is well documented that cannabinoids produce antitumorigenic responses in preclinical models of breast cancer. Cannabinoids such as Δ^9^-tetrahydrocannabinol can have an effect on cancer progression, cell proliferation, survival, angiogenesis, and metastasis (Velasco et al., 2012). While a significant proportion of the actions of cannabinoids are mediated through activation of CB_1_ or CB_2_ receptors, other cannabinoids produce effects that are either completely or partially independent from the cannabinoid receptors and instead act through other targets such as GPR55 and/or GPR18 (Ryberg et al., 2007; Sharir and Abood, 2010; Irving et al., 2017). GPR55 is believed to be the receptor for the phospholipid lysophosphatidylinositol (LPI) (Oka et al., 2007), while GPR18 was suggested to be the receptor for *N*-arachidonylglycine (Kohno et al., 2006). Interestingly, LPI and GPR55 have been associated with cancer progression. Downregulation of GPR55 reduced tumor growth in a xenograft model of glioblastoma and is more resistant to skin carcinogenesis. The LPI/GPR55 axis was also shown to enhance breast cancer cell migration and metastasis (Andradas et al., 2011; Pérez-Gómez et al., 2013; Andradas et al., 2016; Zhou et al., 2018). The endocannabinoid anandamide and other cannabinoids such as the regiosomer of cannabidiol (CBD) (**Figure 1**), known as “abnormal” CBD, and a related compound, O-1602, have been shown to act as agonists of GPR18 and GPR55 receptors (Johns et al., 2007; Kapur et al., 2009; McHugh et al., 2010; McHugh et al., 2012). It is possible that these drugs, just like other compounds activating these receptors, may mediate different effects. Increasing evidence now suggests that ligands acting through these receptors play an important role in the progression of many cancer types (Leyva-Illades and Demorrow, 2013; Falasca and Ferro, 2016; Tegeder, 2016). While it has been shown by other groups that different cannabinoids can reduce the viability of ER^+^/PR^+^ or triple-negative breast cancer cells, it has not been demonstrated that cannabinoids could be useful once these cells become resistant to chemotherapeutics. In this context, we aimed at evaluating the potential antitumorigenic activities of abnormal CBD and O-1602, and examined these effects *in vitro* using paclitaxel-resistant (PR) breast cancer cell lines and *in vivo*, using a zebrafish xenograft model.

## Materials and Methods

### Materials

Paclitaxel and thiazolyl blue methyltetrazolium bromide (MTT) were obtained from Millipore-Sigma and dimethyl sulfoxide (DMSO) from Fisher BioReagents. O-1602 and abnormal CBD were obtained from Cayman Chemical Company. For all experiments, the drugs were dissolved in DMSO. The structures of cannabinoids used in this study were drawn using ChemDraw Prime 16.0.

### Cell Culture

Human breast adenocarcinoma MDA-MB-231 and MCF-7 cells were cultured in Dulbecco modified eagle medium (DMEM)–high glucose (Millipore-Sigma) with 1% penicillin-streptomycin containing 10% fetal bovine serum (FBS) (Gibco, Life Technologies) at 37°C, in a 5% CO_2_ atmosphere. The PR, triple-negative breast cancer cell lines were created from drug-sensitive MDA-MB-231 cells (kindly provided by Drs. David Hoskin and Anna Greenshields, Dalhousie University) as described previously (Hall et al., 2017). The MCF-10A cells were also obtained from Dr. Hoskin. The PR ER^+^, PR^+^, HER2- MCF-7 cells were kindly provided by Drs. Robert Robey and Susan Bates (National Cancer Institute, Bethesda, MD) and were derived from the parental MCF-7 cells by serial passages in increasing concentrations of paclitaxel.

### MTT Assays

Cells were seeded at 20,000 cells/well in 96-well plates and grown for 24 h before adding drugs. Cells were treated with increasing doses of O-1602 or abnormal CBD (0–10 µM; DMSO vehicle control) for up to 48 h. To assess viability, MTT, was added to each well and incubated for 3 h at 37°C before addition of the MTT solvent (4 mM HCl, 0.1% Nonidet P-40, in isopropanol). Absorbance was read at 590 nm with a Biotek Cytation 3 and the quantity of purple formazan quantified as a measure of cell viability. Data are expressed as the percentage of viable cells versus vehicle-treated cells, normalized as 100% and represented as mean ± SEM. The *p* values represent data from at least three independent experiments conducted in quintuplicate.

### siRNAs And Transfections

Negative control siRNA (ID 1027310) was purchased from Qiagen; GPR55-specific (s17761 and ss17760) and GPR18-specific (s223778) siRNAs were purchased from Ambion. Semiconfluent plates were transfected with siRNAs per the manufacturer’s recommendations for 48 h prior to experimentation.

### Cell Lysis and Western Blotting

Cells were lysed with RIPA buffer (150 mM NaCl, 50 mM Tris-HCl pH 7.5, 1% NP4O, 0.5% sodium deoxycholate, 0.1% sodium dodecyl sulfate, and one complete EDTA-free protease inhibitor cocktail tablet; Roche). Bovine serum albumin–coated beads (Protein A-Sepharose; Sigma-Aldrich) and 10% DNase I (Sigma-Aldrich) were added to remove nucleic acid and organellar material from the sample. Lysates were mixed 50:50 with 2× Laemmli buffer and 2-mercaptoethanol (Bio-Rad Laboratories). Samples were run on a sodium dodecyl sulfate–polyacrylamide electrophoresis gel and transferred to nitrocellulose membranes before being blocked in a 10% skim milk powder/phosphate-buffered saline (PBS) solution for 60 min and incubated overnight at 4°C with their respective primary antibodies (GPR55: ab229626 from Abcam, GPR18: TA340675 from Origene, cleaved caspase 3 [p11]: sc-271759 from Santa Cruz Biotechnologies). Chemiluminescence was performed on nitrocellulose membranes using Western Lightning® Plus-ECL Enhanced Chemiluminescence Substrate (PerkinElmer) before exposing them to x-ray film and development.

### Apoptosis Assay

Cells were grown on glass coverslips in six-well plates and then treated with DMSO or 2 µM O-1602 or abnormal CBD in DMSO for 24 h. The annexin V apoptosis detection kit (Santa Cruz Biotechnology) was used to determine the rate of apoptosis. Cells were harvested and washed with PBS and then resuspended in annexin V assay buffer following the manufacturer’s instructions. Cells were gently shaken in the dark with propidium iodide (PI) and annexin V–fluorescein isothiocyanate–conjugated stain for 20 min. Cells were then examined by fluorescence microscopy, and at least five fields of view were recorded using an Olympus IX81 microscope equipped with a Photometrics coolSNAP HQ2 camera and an Excite series 120Q light source. Annexin V stain was excited at 488 nm, and images were captured at 525 nm. Propidium iodide was excited at 535 nm and images captured at 617 nm. Rates of early apoptosis were determined by dividing the number of cells that stained positive for annexin V divided by the total number of cells (Young et al., 2015; Martin et al., 2019).

### Reactive Oxygen Species Measurements

Forty thousand cells were plated in a 96-well black plate in 100 μl of DMEM 1% FBS per well and incubated at 37°C and 5% CO_2_ for 4 h to allow the cells to adhere to the plate. After incubation, the transfected cells were stimulated with O-1602 or Abn-CBD, or vehicle. After 24 h, each well was washed twice with 1× PBS. Next, 100 μl of 1× Hanks balanced salt solution (HBSS) was added to each well. CM-DCFH2-DA (Life Technologies Corp.) in DMSO at 40 μM was added to each well for 1 h at 37°C. After the incubation, the cells were washed twice with PBS, and 100 μl of HBSS was added to each well. The fluorescence of the wells was then read on a BioTek Cytation 3 plate reader using the monochromators at 485 nm for excitation and 528 nm for emission.

### Transwell Migration Assays

Paclitaxel-resistant MDA-MB-231 cultures were prepared such that 5.0 × 10^5^ cells/condition were resuspended in DMEM and plated into the top portion of a Transwell migration plate that contains a polycarbonate membrane with a pore size of 5.0 µm (Costar). In the bottom portion of the well, 600 µL of DMEM containing 10 ng/ml of epidermal growth factor (EGF), a potent activator of cell migration, was added. Cells were incubated for 24 h in the presence of various concentrations of O-1602 or abnormal CBD (0–1 µM) for their ability to change breast cancer cell migration patterns. For quantification, membranes were rinsed with cold PBS and fixed in 100% ice-cold methanol for 15 min at room temperature. Fixed membranes were then stained with 0.5% crystal violet stain for 5 min to allow for visualization of the cells. Nonmigrated cells were then gently removed from the upper side of the membrane with a cotton bud. Membranes were rinsed in dH_2_O until the water ran clear, allowed to dry, and then mounted on a slide. At least three areas of the membrane were viewed under the 10× objective of an Olympus IX81, and the number of cells for each field of view was counted. Net migration was determined by comparing the number of cells that migrated with the chemoattractant to the number of cells that migrated under control conditions (DMSO).

### Invasion Assay Protocol

Growth factor reduced 8.0-µm Matrigel Invasion Chambers (Corning), and Cell Culture Inserts with an 8.0-µm membrane were added to a 24-well plate. Matrigel Invasion Chambers were hydrated with 250 µL of DMEM-high glucose containing 0.2% FBS, penicillin-streptomycin, and 2 µM of O1602, abnormal CBD, or vehicle control and were then incubated for 1 h at 37°C. Paclitaxel-resistant MDA-MB-231 cells were then seeded in DMEM without FBS at a concentration of 100,000 cells/ml. After 1 h, 700 µL of DMEM containing 10% FBS was added to the lower chamber of invasion chambers and cell culture inserts. Two hundred fifty microliters of DMEM containing 0.2% FBS and DMSO was added to the cell culture inserts, and then 250 µl of the cell suspension was added to each cell culture insert and Matrigel invasion chamber resulting in a final drug concentration of 1 µM at 37°C. After 24 h, media and cells that did not migrate were removed from the inside of the insert. Wells were placed in methanol for 10 min and then transferred into a 3.5 g/L crystal violet in 2% ethanol solution for 10 min. Wells were then rinsed with H_2_O and left to dry overnight. Cells that migrated or invaded through the membranes were counted using an Olympus CKX41 light microscope. Percent invasion was calculated by dividing the number of cells invaded in each condition by the number of cells migrated in the control.

### Larval Zebrafish Rearing

Zebrafish (*Danio rerio*) were maintained according to standard animal care protocols and in accordance with the Canadian Council on Animal Care guidelines. AB/Tubingen adults, embryos, and larvae were maintained on a recirculating Tecniplast aquatic system at 28.5°C ± 0.5°C and between pH 7.0 and 7.5 on a 14/10-h light/dark (L/D) cycle. Embryos were collected from multiple AB/Tubingen breeding pairs and pooled. Following 4 to 6 h in an incubator in E3 media (5 mM NaCl, 0.17 mM KCL, 0.33 mM CaCl_2_·2H_2_O, 0.33 mM MgSO_4_), unfertilized embryos were removed. Larvae were placed in Aquatic Habitats mesh-bottom baby baskets on the recirculation system until use (maximum 200 embryos per basket) residing in a 3-L tank in a ZebTec Recirculation Water Treatment System (Tecniplast, USA).

### Drug Toxicity

At 48 h postfertilization (hpf), embryos were manually dechorionated using Dumont #3 forceps and rinsed in HEPES-buffered E3 media (HE3) (10 mM, HEPES pH 7.2, 5 mM NaCl, 0.17 mM KCl, 0.33 mM CaCl_2_·2H_2_O, 0.33 mM MgSO_4_). Embryos were then loaded into a 96-well plate, 1 embryo/well with 270 µL HE3/well using a large-bore micropipette tip created by cutting off the tip. A 10× solution of O-1602 or abnormal CBD was subsequently added to each well at final exposure concentrations up to 10 µM. Final DMSO concentration was held at 0.5% (v/v) at all dilutions of drugs tested. Plates were then placed in an incubator at 34°C and assessed at 120 hpf for both developmental abnormalities and death. Replicate experiments were run on separate days (n = 12 per day).

### Xenograft

Five million taxol-resistant MDA-MB-231 cells were pelleted by centrifugation (5 min at 100*g*), resuspended in PBS containing CM-DiI (ThermoFisher Scientific) (5 µg/ml), and incubated for 5 min at 37°C and 20 min at 4°C. Cells were pelleted, washed twice with PBS, and resuspended in DMEM. Dechorionated 48-h zebrafish embryos were anesthetized with tricaine and 75 to 150 labeled cells injected into the yolk sac (adapted from (Haldi et al., 2006). Following 1-h recovery at 28°C, embryos were screened for fluorescence at the injection site. O-1602 or abnormal CBD (2 µM) or vehicle (DMSO) was added to the water of injected embryos. Embryos were maintained at 34°C, and drug treatments were repeated daily for 72 h. Proliferation of human breast cancer cells was monitored by assessing fluorescence using live-cell microscopy.

### Statistical Analysis

Statistical analysis was completed using GraphPad Prism software. All error bars are representative of mean ± SEM. Unpaired Student *t* tests were performed for analysis of two independent groups. One-way analysis of variance with Tukey *post hoc* test was used to assess multigroup comparisons. *p* values are reported as follows: *p < 0.05, **p < 0.01, ***p < 0.001.

## Results

### Cell Viability

**Figure 2:**
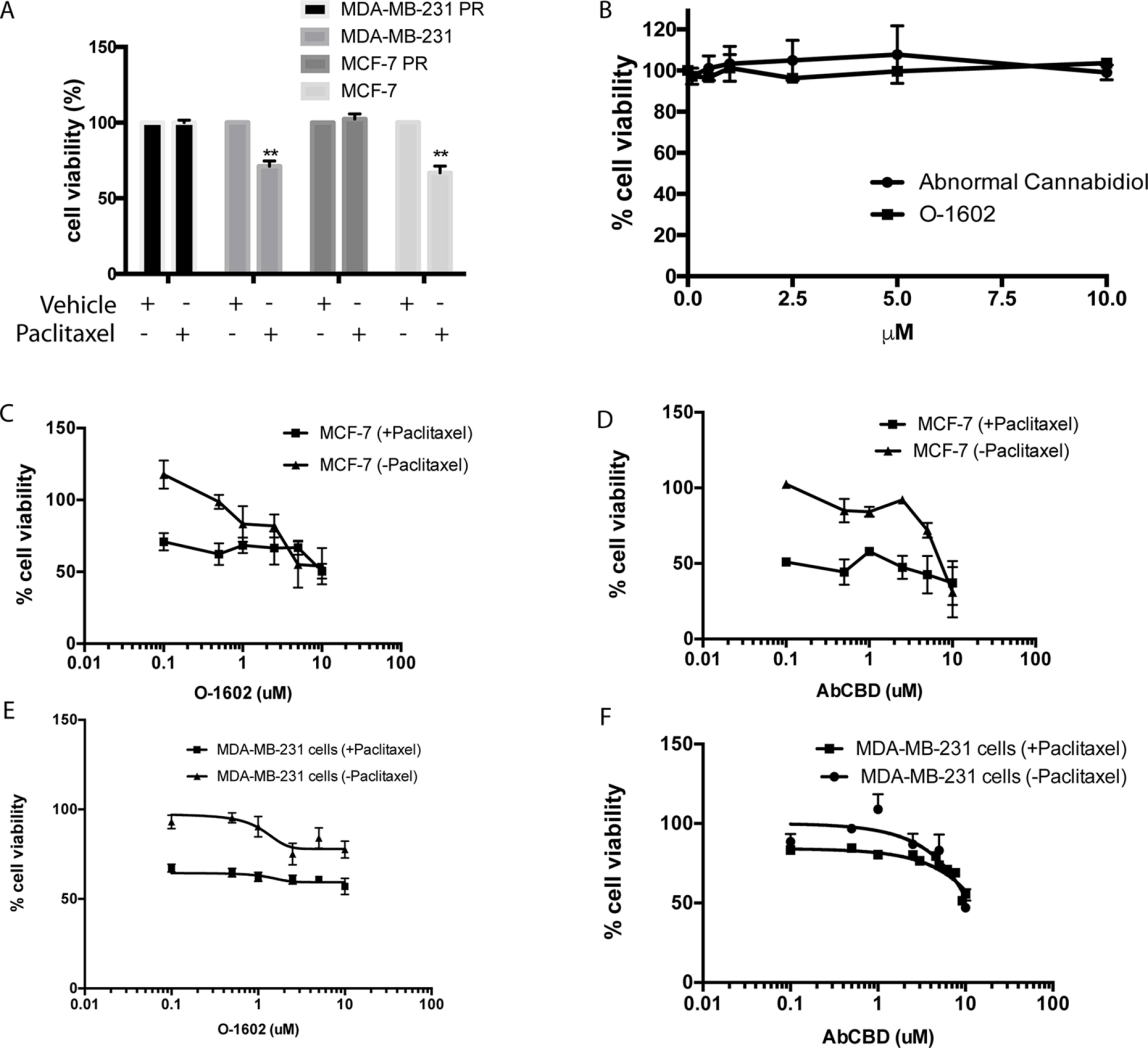
Cell viability. **(A)** Effect of paclitaxel on sensitive and resistant (PR) MCF-7 and MDA-MB-231 cells. **(B)** Effect of O-1602 and Abn-CBD on the cell viability of MCF-10A cells. **(C)** Cell viability measured in paclitaxel-sensitive MCF-7 cells following 48-h treatment with various concentrations of O-1602 alone or in presence of paclitaxel. **(D)** Effect of Abn-CBD ± paclitaxel on taxol-sensitive MCF-7 cells. **(E)** Effect of O-1602 ± paclitaxel on taxol-sensitive MDA-MB-231 cells. **(F)** Effect of Abn-CBD ± paclitaxel on taxol-sensitive MDA-MB-231 cells.

One commonly used chemotherapeutic is paclitaxel, because of its effects on cytoskeletal rearrangements during cell replication and migration. Here, we use paclitaxel-sensitive and PR MCF-7 cell lines to identify whether cannabinoids could be effective against breast cancer cell lines and chemotherapeutic-resistant breast cancer cells. Due to the apparent importance of their receptors in certain cancer types, two atypical cannabinoids, abnormal CBD (Abn-CBD) and O-1602 that mediate their effects through GPR55 and GPR18, were selected in our study. First, we show that the cell lines used in our study are indeed sensitive or resistant to paclitaxel (470 nM). This concentration of paclitaxel kills nonresistant cells but leaves the resistant cell line unaffected in terms of viability (Issa et al., 2014) (**Figure 2A**). Then, several reports suggest that cannabinoids can kill cancer cells at concentrations that leave noncancer cell lines unaffected. We confirm that this is the case for O-1602 and Abn-CBD in MCF-10A cells (**Figure 2B**). Then, we show the effects of various concentrations of O-1602 and Abn-CBD ranging from 0.1 to 10 µM, alone or in addition to 470 nM paclitaxel for 48 h on breast cancer cell lines that are not resistant to paclitaxel. **Figure 2C–F** show the concentration dependent effect of O-1602 and Abn-CBD on MCF-7 and MDA-MB231 cells. Addition of paclitaxel to these cells shows that paclitaxel can induce cell death, but no synergistic effect is observed when O-1602 or Abn-CBD is added concomitantly (**Figures 2C–F**).

**Figure 3:**
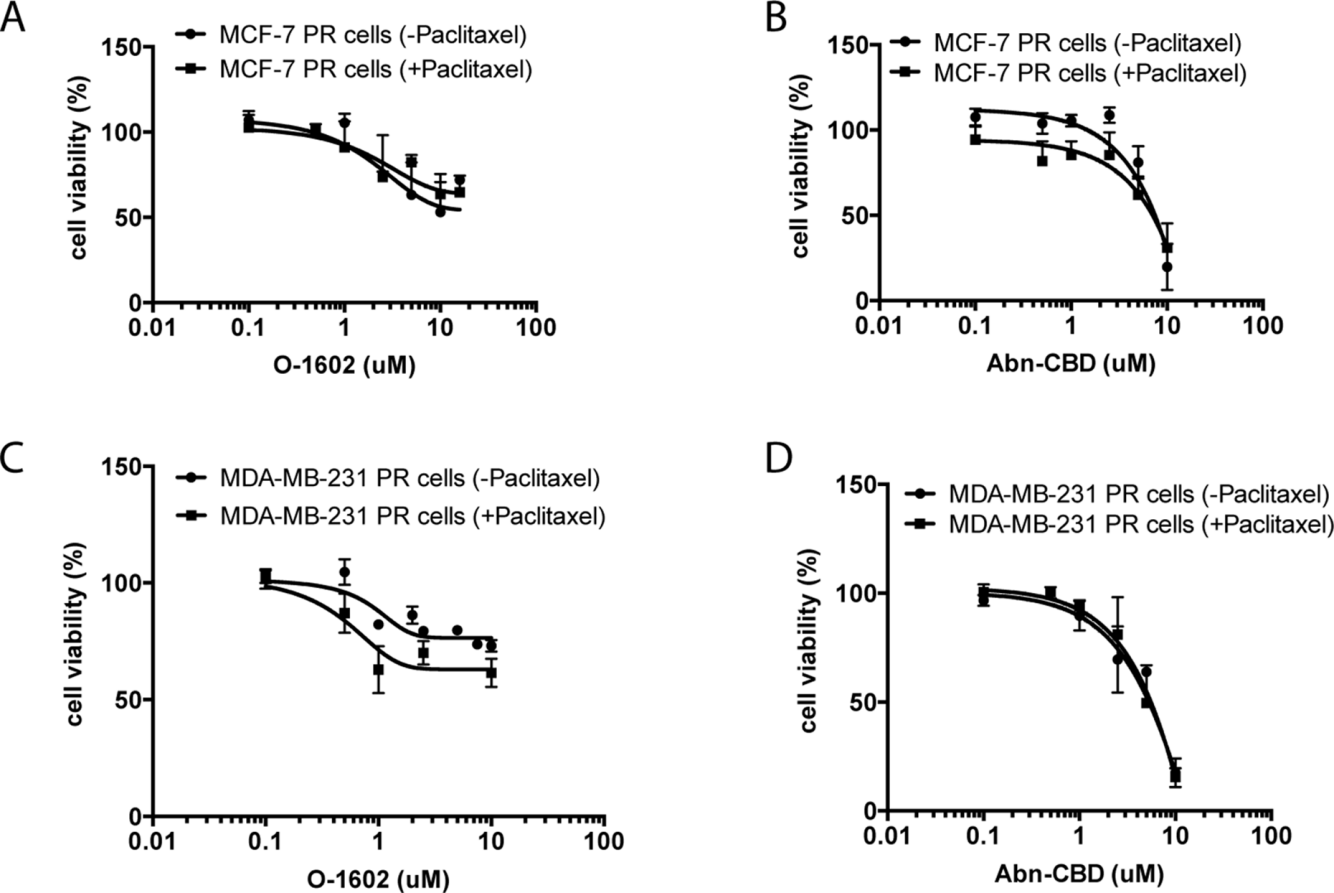
Atypical cannabinoids effect on the viability of paclitaxel-resistant cells. **(A)** Cell viability measured in paclitaxel-resistant (PR) MCF-7 cells following 48-h treatment with various concentrations of O-1602 alone or in presence of paclitaxel. **(B)** Effect of Abn-CBD ± paclitaxel on MCF-7 PR cells. **(C)** Effect of O-1602 ± paclitaxel on MDA-MB-231 PR cells. **(D)** Effect of Abn-CBD ± paclitaxel on MDA-MB-231 PR cells.

We then proceeded to evaluate the effect of the same concentrations of O-1602 alone, or in addition to 470 nM paclitaxel for 48 h on paclitaxel-resistant cell lines (**Figure 3**). No significant differences were observed between the curves of O-1602 alone or O-1602 with paclitaxel. Similarly, the response to 0.1 to 10 µM Abn-CBD alone was generally comparable to the response obtained in addition to paclitaxel. At lower concentrations of Abn-CBD, the viability of the paclitaxel-resistant MCF-7 was trending toward a greater effect when combined with paclitaxel, but this effect was lost at higher concentrations of Abn-CBD. Next, we evaluated the effects of cannabinoids on the triple-negative MDA-MB-231 cell line. Again, the cells were incubated in the presence of various concentrations of O-1602 ranging from 0.1 to 10 µM (**Figure 3**) alone or in combination with 470 nM paclitaxel. O-1602 again decreased the paclitaxel-resistant cell viability and the addition of paclitaxel to the treatment slightly increased the effect, albeit not significantly. The response to 0.1 to 10 µM Abn-CBD alone or in addition to paclitaxel was not significantly different. Our results suggest that paclitaxel-resistant breast cancer cells are susceptible to the antitumor effects of these cannabinoids.

#### Receptor Dependency

**Figure 4:**
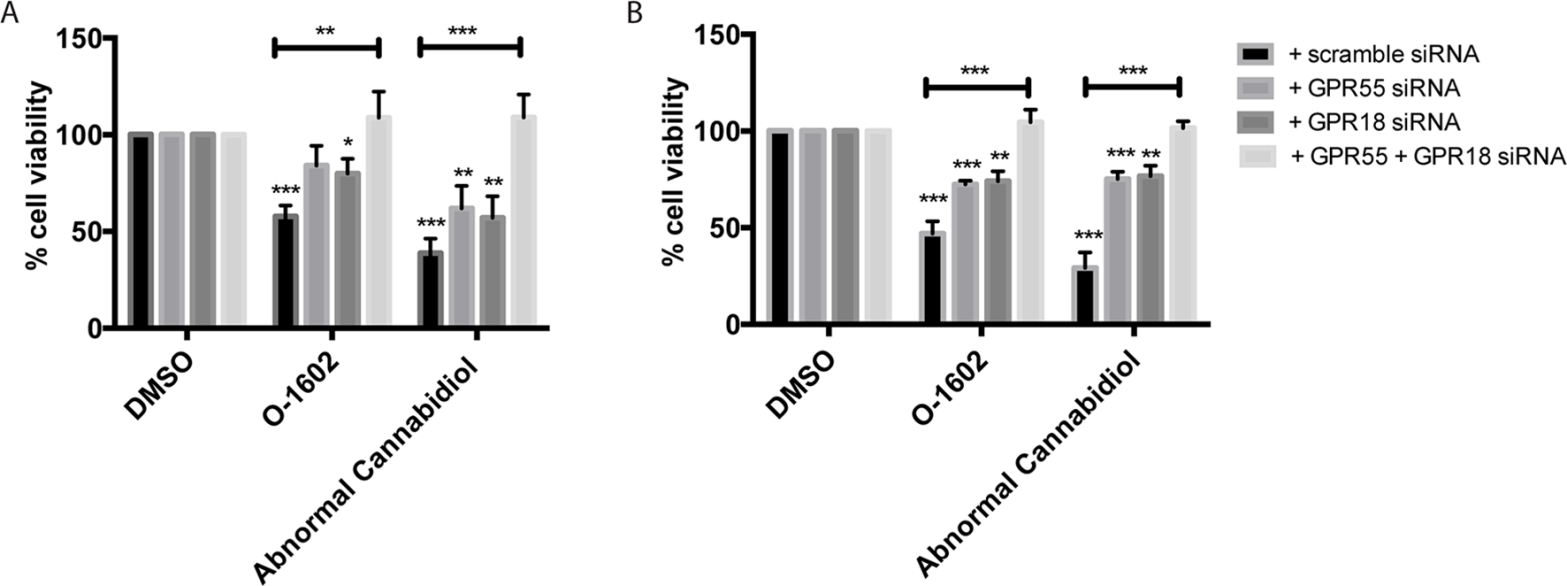
Receptor dependence of effect on cell viability. **(A)** Effect of the GPR55-, GPR18-specific small interfering RNAs (siRNAs), or combination of siRNAs for both targets on cell viability in **(B)** MDA-MB-231 PR cells and (B) MCF-7 PR cells. *p < 0.05, **p < 0.01, ***p < 0.001.

Next, we aimed to identify the target responsible for the cytotoxic effects of O-1602 and Abn-CBD. These atypical cannabinoids have generally been associated with binding and activation of two G protein–coupled receptors, GPR55 and GPR18. Thus, we transfected paclitaxel-resistant MDA-MB-231 cells with GPR55 or GPR18 siRNAs to help determine their effect on the cannabinoids’ activity. First, the siRNAs strongly diminished the expression of the receptors, but their effect is not complete for GPR18 (**Supplementary Data**). Then, siRNA-expressing cells were treated with 2 µM of either O-1602 or Abn-CBD for 48 h, and a cell viability assay was performed. Our results indicate that in the presence of the control scramble siRNA we observed a decrease of approximately 50% in cell viability of the paclitaxel-resistant MDA-MB-231 cells (**Figure 4A**). When either the GPR55 or the GPR18 siRNAs were expressed alone, the drugs’ effects were partially blocked. O-1602 and Abn-CBD’s effects were completely abolished in the presence of siRNAs for both targets simultaneously. Similar effects were observed with the paclitaxel-resistant MCF-7 cells (**Figure 4B**).

### Apoptosis Assay

**Figure 5:**
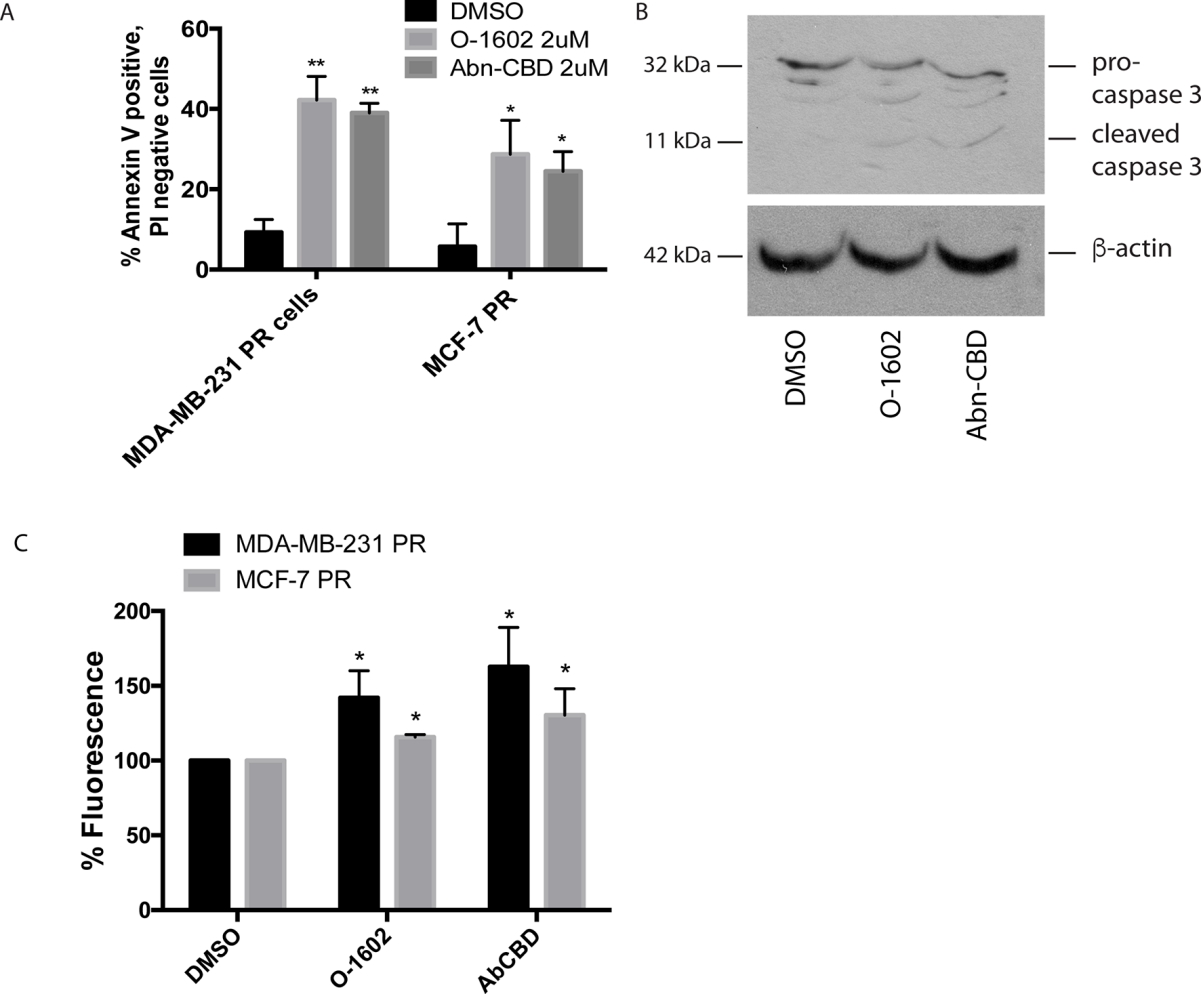
Effect of atypical cannabinoids on apoptosis. Cells were treated for 24 h with either the DMSO vehicle, O-1602, or Abn-CBD. **(A)** Histogram showing the % of annexin V–labeled cells. Cells staining for PI only or both PI/AV are not shown. Cells were counted from three random fields of view on a fluorescence microscope. *p < 0.05, **p < 0.01, n = 3. **(B)** Western blotting analysis was performed using an anti–caspase-3 antibody, and β-tubulin was included as a loading control. Figure is a representative blot of n = 3 experiments. **(C)** Reactive oxygen species assay measurements in MDA-MB-231 and MCF-7 cells following treatment with O-1602 or Abn-CBD. (n = 3).

We next determined whether the different drug treatments were inducing cell death *via* apoptosis using annexin V staining and PI. In all drug treatments, approximately 5% of cells were stained with PI staining (data not shown). **Figure 5A** shows the effects of a 24-h treatment with 2 µM O-1602 or Abn-CBD on annexin V labeling of paclitaxel-resistant MCF-7 and MDA-MB-231 cells. Approximately 10% of cells were positive for annexin V in response to the vehicle control DMSO. Meanwhile, approximately 40% of MDA-MB-231 cells were positive for annexin V, while 25% to 30% of MCF-7 cells were labeled, in response to O-1602 and Abn-CBD, respectively. **Figure 5B** shows the cleavage of the proapoptotic protein caspase 3 in response to the treatment of MDA-MB-231 cells with O-1602 or Abn-CBD, at 2 µM for 24 h. The antitumor activities of cannabinoids have been characterized in various cell lines and involve autophagy, AMPK, and/or reactive oxygen species (ROS). We demonstrate in **Figure 5C** that O-1602 and to a greater extent Abn-CBD can induce ROS production in both paclitaxel-resistant breast cancer cell lines.

### *In Vitro* Migration and Invasion

**Figure 6:**
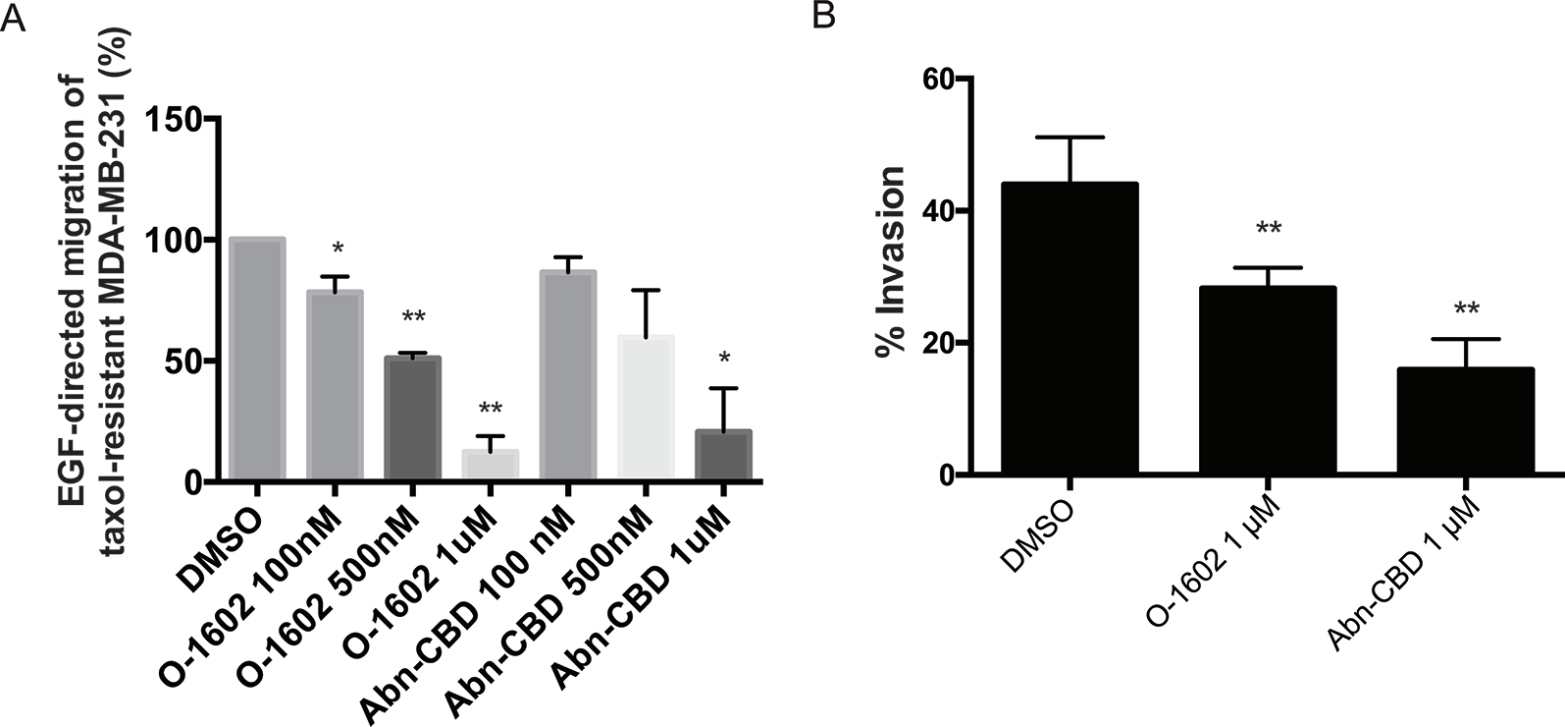
Effect of atypical cannabinoids on EGF-mediated migration and invasion. **(A)** Histogram summarizing Transwell migration results using MDA-MB-231 PR cells in response to EGF in presence of either the vehicle control or various concentrations of O-1602 or Abn-CBD. **(B)** Histogram summarizing Matrigel invasion using MDA-MB-231 PR cells in presence of either the vehicle control, O-1602, or Abn-CBD. Results represent the means ± SEM of at least three independent experiments. *p < 0.05, **p < 0.01.

As previously mentioned, one of the features of triple-negative breast cancer cells is their aggressiveness with respect to migration. We used paclitaxel-resistant MDA-MB-231 cells to assess the effects of O-1602 and Abn-CBD on the migratory properties of this cell line using a Transwell assay. Epidermal growth factor at 10 ng/ml was used as a potent inducer of cell migration. Cells were treated for 6 h with EGF in presence of DMSO (control) or various concentrations of O-1602 or Abn-CBD. Our results indicate that in comparison to the control, a concentration-dependent effect was observed for the inhibition of migration by O-1602 and Abn-CBD (**Figure 6A**). A 22% decrease was observed following treatment with 100 nM of O-1602, 49% decrease with 500 nM, and 88% decrease with 1 µM. For Abn-CBD, 14%, 41%, and 80% decreases were observed, at 100 nM, 500 nM, and 1 µM, respectively. Similarly, when the paclitaxel-resistant MDA-MB-231 cells were used for an invasion assay, we observed that O-1602 and Abn-CBD decreased the levels of invasion in comparison to the vehicle control DMSO (**Figure 6B**).

### *In Vivo* Toxicity

**Figure 7:**
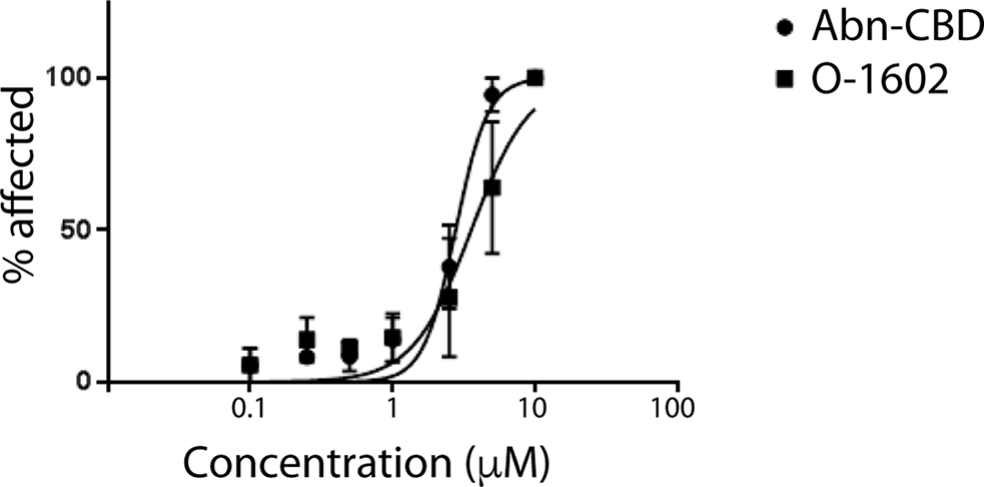
Toxicity of atypical cannabinoids in zebrafish. Graph showing the percentage of zebrafish larvae affected by various concentrations of O-1602 or Abn-CBD in water for 3 days.

The developmental toxicity/teratogenicity of each cannabinoid in our study was tested by continuous exposure of zebrafish embryos to either O-1602 or Abn-CBD at various concentrations between 48 and 120 hpf. Our results indicate that phenotypic abnormalities associated with each drug tested were apparent in a concentration-dependent fashion, including developmental delay, malformations, and truncated tail. EC_50_ values were calculated to compare toxicity levels between each drug by measuring the percentage of larvae that showed one or more phenotypic abnormality. The results were used to generate a concentration-response curve based on the percentage of affected larvae at each concentration. Our results indicate that concentrations greater than 2.5 µM displayed higher levels of toxicity to the larvae (**Figure 7**).

### *In Vivo* Cell Viability

**Figure 8:**
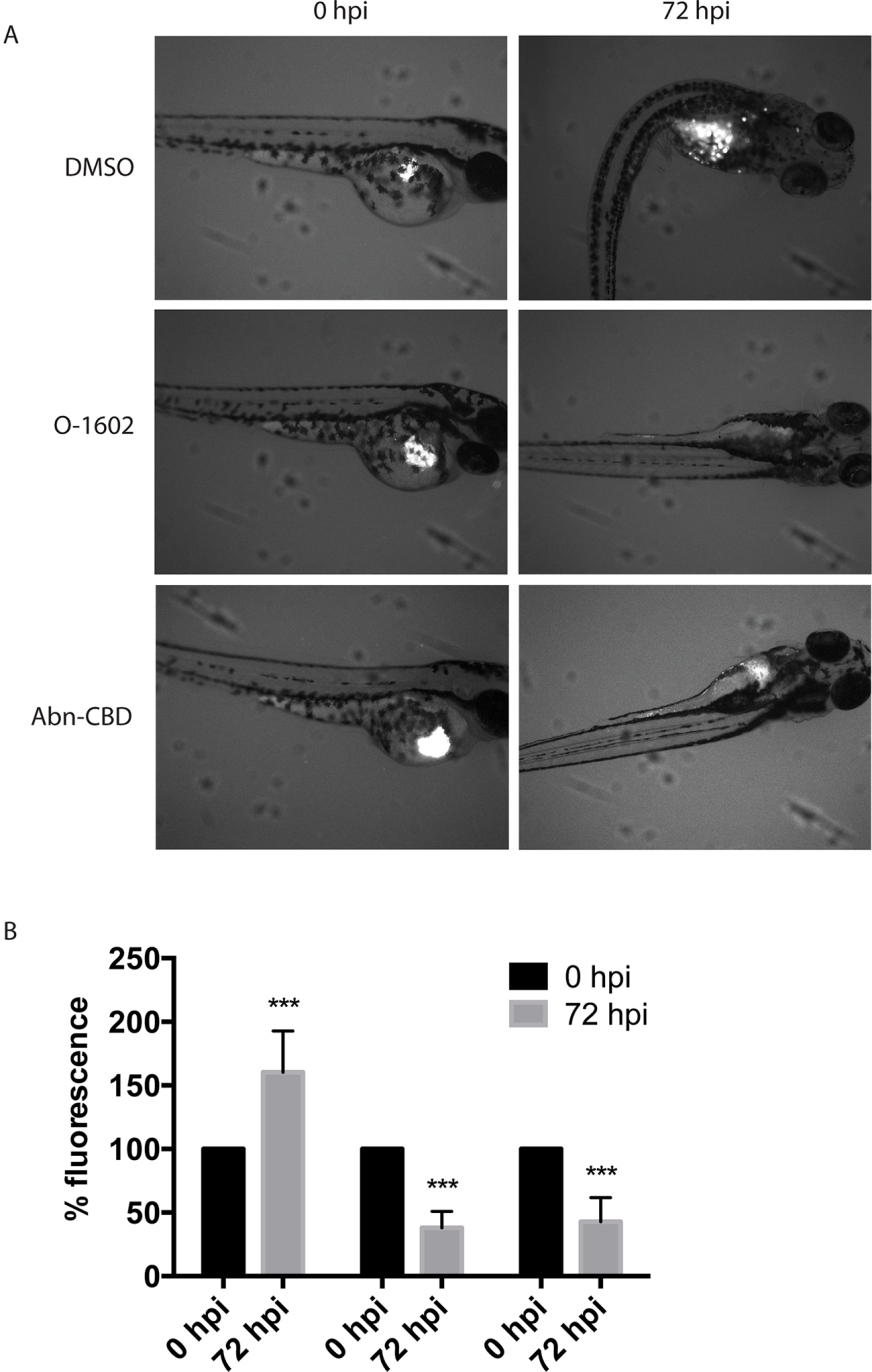
Effect of atypical cannabinoids on the viability of Paclitaxel-resistant breast cancer cells *in vivo*. **(A)** Images of representative zebrafish injected with human MDA-MB-231 PR cells in the yolk sac (white pixels) before (0 hpf) and following 3-day treatment with the DMSO vehicle control, O-1602, or Abn-CBD (72 hpf). **(B)** Quantification of the analysis of more than 24 images for each condition. ***p < 0.001.

At 48 hpf, cells labeled with Cm-DiI were injected into the yolk sac of the zebrafish larvae to assess whether the atypical cannabinoids could also exert their antitumor effects *in vivo*. Our results show that incubation of the larvae in fishwater containing 2 µM of O-1602 or Abn-CBD significantly reduces the viability of the injected human paclitaxel-resistant MDA-MB-231 cell line (**Figure 8**). The control larvae permitted an increase in proliferation of the cells in the yolk sac and other areas of the zebrafish body (visible as white spots in **Figure 8A**, quantified in **Figure 8B**); both atypical cannabinoids significantly reduced the presence of injected cancer cells in the zebrafish larvae by approximately 50%, as shown in **Figure 8A**, and quantified in **Figure 8B**.

## Discussion

Many G protein–coupled receptors (GPCRs) have been associated with tumor progression and metastasis. Changes in expression levels or mutation of many GPCRs have been associated with various cancers (Dorsam and Gutkind, 2007) including several receptors that bind cannabinoids. For example, GPR55 expression has been detected in breast cancer cell lines and interestingly was much more abundantly expressed in MDA-MB-231 cells compared with the less metastatic MCF-7. Also, higher expression levels of GPR55 were observed in tumors with worse prognosis (Andradas et al., 2016). Most studies related to the effects of GPR55 have been performed with LPI, proposed as GPR55’s endogenous ligand. In breast cancer, LPI treatment of MDA-MB-231 cells enhances cell chemotaxis, which was prevented by transfection with GPR55 small interfering RNA (Ford et al., 2010). Yet, while LPI seems to contribute to cancer progression and metastasis through the activation of GPR55, it was shown in a cholangiocarcinoma model that an endocannabinoid, anandamine (AEA), whose effects were not mediated through the activation of the typical CB_1_/CB_2_ receptor or vanilloid 1 receptor, suppressed cholangiocarcinoma growth by inducing apoptosis. In fact, in that model, reduced expression of GPR55 abolished the antiproliferative and the growth-suppressing effects of GPR55 activation by AEA (DeMorrow et al., 2007; DeMorrow et al., 2008; Frampton et al., 2010). Interestingly, not only was the treatment with AEA able to reduce cholangiocarcinoma cell proliferation *in vitro* and *in vivo*, but this effect was also observed with another GPR55 agonist, O-1602 (Huang et al., 2011). In our study, we observed that treatment of breast cancer cell lines with the cannabinoids Abn-CBD and O-1602 also reduced cell viability and the ability of the metastatic MDA-MB-231 cells to migrate toward a chemotactic signal. This suggests that while LPI and some cannabinoids activate the same receptors, the effects of cannabinoids may be different from the endogenous ligand, potentially through activation of biased signaling pathways or other yet nonidentified mechanisms. Other receptors, such as GPR18 and GPR35, have also been identified as modulators of tumor progression (Okumura et al., 2004; Qin et al., 2011), but the actual effect of cannabinoids on these receptors is still unclear. In our study, we show that GPR18, just as for GPR55, contributes to the effects of cannabinoids observed on paclitaxel-resistant breast cancer cell viability. Characterization of these receptors will help understand their potential impact on tumor development and progression.

Some studies have proposed cannabinoids as possible adjuvants during cancer therapy partly for their ability to reduce drug resistance (Holland et al., 2006; Holland et al., 2007). In agreement with this notion, a recent report showed that a synthetic cannabinoid, WIN55,212-2, supported the antimyeloma activity of dexamethasone and melphalan, to reduce melphalan resistance in cell culture (Barbado et al., 2017). The phytocannabinoid CBD, structurally similar to the two atypical cannabinoids used in our study, improved the sensitivity of glioblastoma cells to the chemotherapeutic agents carmustine, temozolomide, doxorubicin, and cisplatin (Nabissi et al., 2013; Nabissi et al., 2016; Deng et al., 2017). Synergistic proapoptotic effects were also reported for the combination of the chemotherapeutic agent paclitaxel and AEA in gastric cancer cells (Miyato et al., 2009). While our results do not show synergistic effects between O-1602 or Abn-CBD and paclitaxel, our results still suggest that when paclitaxel is unable to destroy the resistant cancer cells, the cannabinoids are able to induce cell death *via* apoptosis possibly through ROS activation. Other potential pathways like AMPK and autophagy were not evaluated in this study. Combined, those results indicate a significant potential of cannabinoids as possible complementary approach to the current chemotherapeutic treatment of multiple cancer types.

As previously reported in previous studies using breast cancer cell lines (Caffarel et al., 2006; Guindon and Hohmann, 2011; Caffarel et al., 2012; Blasco-Benito et al., 2018), the breast cancer subtypes used in our study were sensitive to cannabinoids, including the highly aggressive triple-negative cancer cell line. Cannabinoids have been shown to display antitumor effects on a variety of cancer cell types, including breast, skin, pancreas, liver or lung adenocarcinomas, glioblastomas, sarcomas, and several others. The variety of these cancer types, combined with the observation that cannabinoids do not appear to display toxic effects on normal cells at the concentrations required to kill cancer cells, suggests cannabinoids potentially tackle a pathway, yet unidentified, that is required for cancer cells to thrive, but is absent or nonessential in normal cells. In our study, paclitaxel-resistant cell lines were used to determine whether cannabinoids could be beneficial in cancers that are unresponsive to chemotherapy, and our results do suggest that these drugs have potential in a therapeutic regimen *in vitro* and *in vivo*. Interestingly, there does not appear to be any resistance mechanism identified permitting cancer cells to evade the actions of cannabinoids, making this class of drug an interesting new therapeutic avenue to explore.

Most studies up to now have used mice for preclinical *in vivo* studies to assess the anticancer effects of cannabinoids. Here, we used zebrafish (*D. rerio*) as our screening model for drug effect on cancer viability *in vivo*. Phylogenetic studies demonstrate that the endocannabinoid system is highly conserved between zebrafish and mammals and that expression of this system begins early in the zebrafish development (McPartland et al., 2007). Zebrafish absorb small molecules from the water at all stages of development, making them an excellent tool for drug screening. Other characteristics, such as their rapid development outside the mother, transparency at embryonic and larval stages, and the high fecundity of adult mating pairs leading to hundreds of offspring each week allowing for live imaging and cost-effective compound screening make the zebrafish model an attractive complementary model to more classical mouse models (Kirchberger et al., 2017). Other advantages for xenograft studies include that the recipient embryos do not require conditioning prior to transplant as zebrafish embryos do not have a fully developed immune system (Nicoli et al., 2007), easy visualization *via* fluorescent tagging of tumor cells, and large numbers of embryos can be transplanted for studies, and only a small number of cells is required for each xenograft (Hill et al., 2018). While it does not replace mice as a model for xenograft, zebrafish represents a rapid, cost-effective way to screen drugs for their anticancer properties.

Our results suggest that some cannabinoids acting through the GPR55 and/or GPR18 receptors can be helpful in inducing apoptosis in breast cancer cell lines that are unresponsive to paclitaxel. The effects of O-1602 and Abn-CBD on cell viability were observed both *in vitro* and in a zebrafish xenograft model. These drugs were also reducing cell migration. Taken together, even if no synergistic antitumor effect is always observed when cannabinoids and chemotherapeutic agents are combined as an anticancer treatment, cannabinoids can still provide anticancer benefits on top of their palliative effects. This is particularly important in the context of cancers that have developed resistance to current chemotherapies.

## Data Availability Statement

The datasets generated for this study are available on request to the corresponding author.

## Ethics Statement

The animal husbandry and larval zebrafish rearing was approved by the National Research Council’s animal care committee.

## Author Contributions

AT, HT, LO’L, and DD contributed conception and design of the study and performed the *in vitro* work. JA, LE, and DD designed and performed the *in vivo* work. SH and KG established and characterized the taxol-resistant breast cancer cell lines. All authors contributed to manuscript revisions and read and approved the submitted version.

## Funding

The work for this study was supported by the Beatrice Hunter Cancer Research Institute Seed Funding to DD.

## Conflict of Interest

The authors declare that the research was conducted in the absence of any commercial or financial relationships that could be construed as a potential conflict of interest.
