# Supplementary figures and images for: Antitumor Activity of Abnormal Cannabidiol and Its Analog O-1602 in Taxol-Resistant Preclinical Models of Breast Cancer
Source: Front Pharmacol. 2019 Sep 27;10:1124. doi: 10.3389/fphar.2019.01124 (PMC6777324; doi:10.3389/fphar.2019.01124)

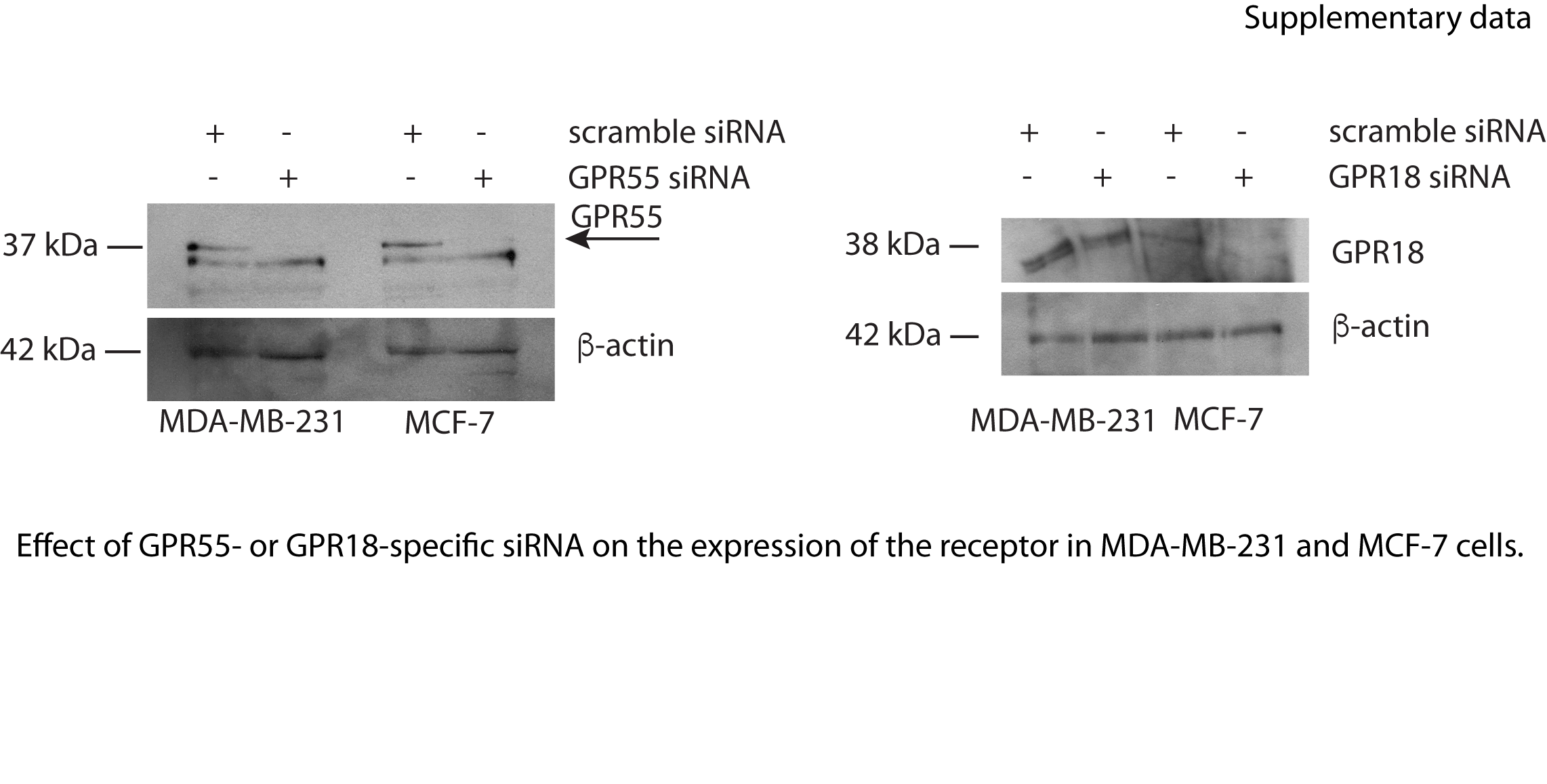

Supplement: Supplementary file 1 [file Image_1.tif]
